# Supplementary material for: Vitamin D Status and Reference Intervals Measured by Liquid Chromatography–Tandem Mass Spectrometry for the Early Adulthood to Geriatric Ages in a South Korean Population during 2017–2022
Source: Nutrients. 2024 Feb 22;16(5):604. doi: 10.3390/nu16050604 (PMC10934696; doi:10.3390/nu16050604)
Supplement: Supplementary file 1 [file nutrients-16-00604-s001.zip › nutrients-2873283-supplementary.pdf]

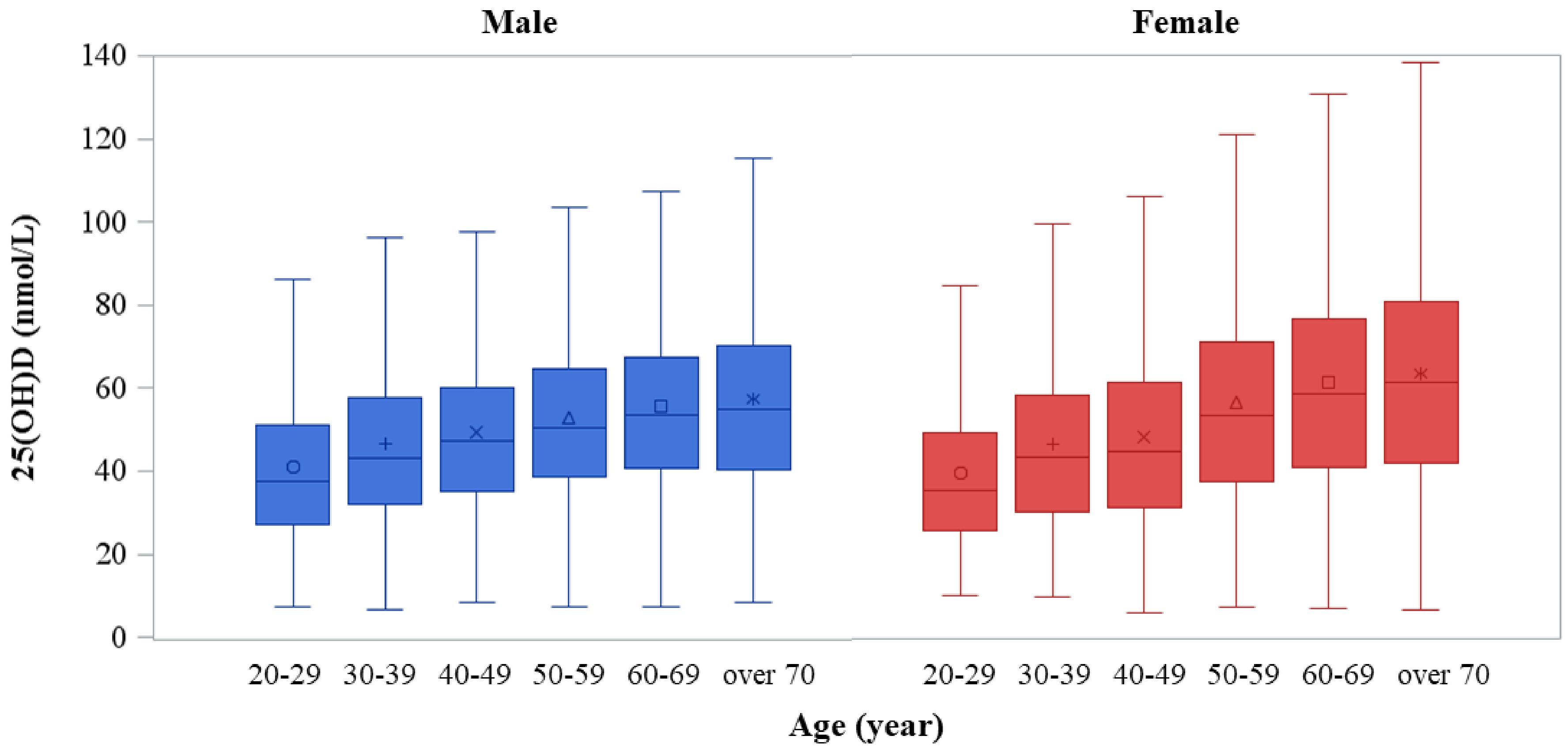

Supplementary Figure S1. Distributions of serum 25(OH)D concentrations by age and sex. The upper and lower whiskers indicate the 97.5th and 2.5th percentiles, respectively. For each sex, comparison of median values among age groups was performed using the Kruskal-Wallis test, and pairwise comparisons between adjacent age groups were performed using the Dwass-Steel-Critchlow-Fligner method (P<0.05). 25(OH)D, 25-hydroxyvitamin D

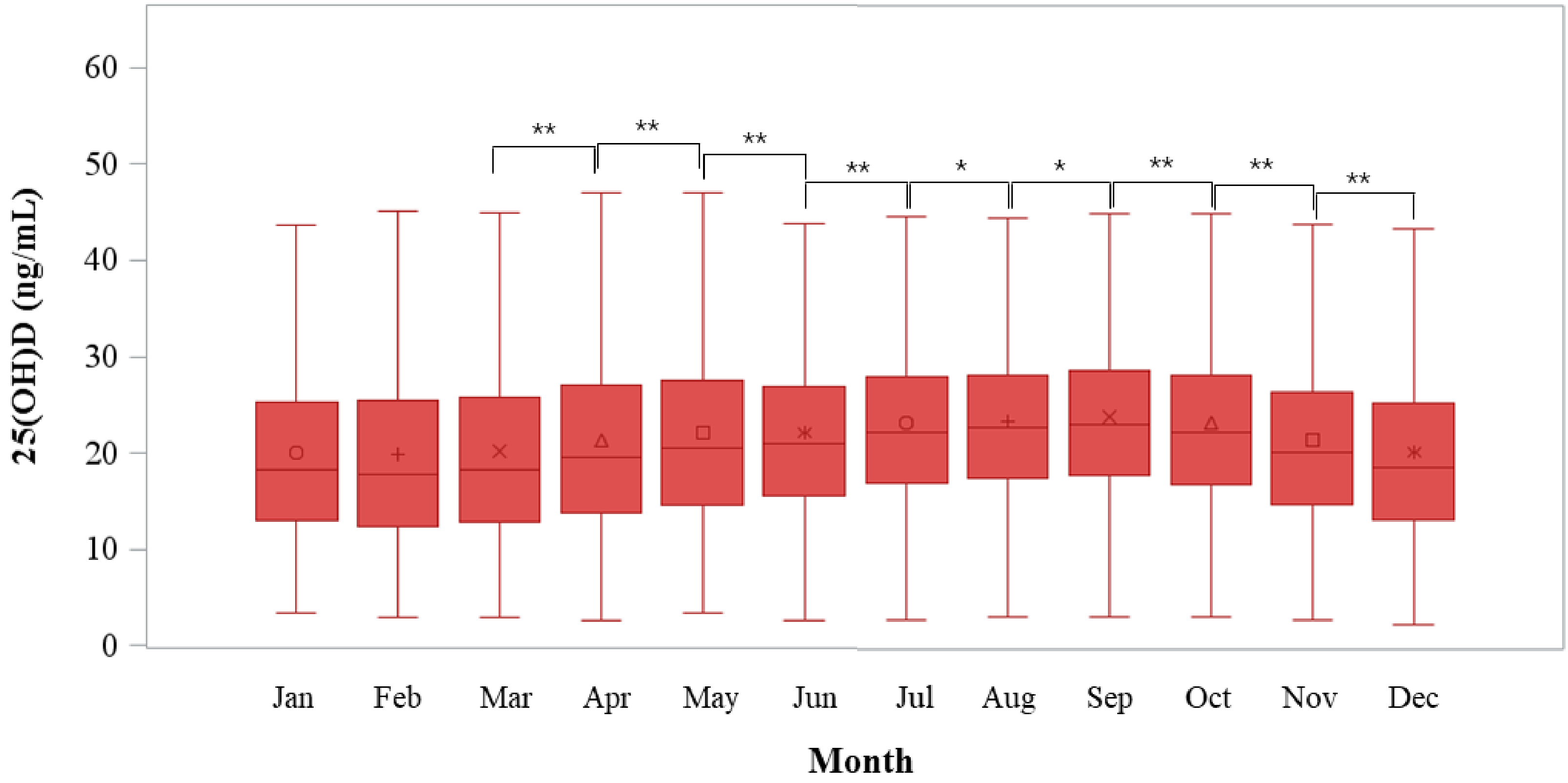

Supplementary Figure S2. Distributions of serum 25(OH)D concentrations by month of checkup. The upper and lower whiskers indicate the 97.5th and 2.5th percentiles, respectively. Comparison of median values among sampling month was performed using the Kruskal-Wallis test, and pairwise comparisons between adjacent months were performed using the Dwass-Steel-Critchlow-Fligner method (**P*<0.05, ***P*<0.01). 25(OH)D, 25-hydroxyvitamin D

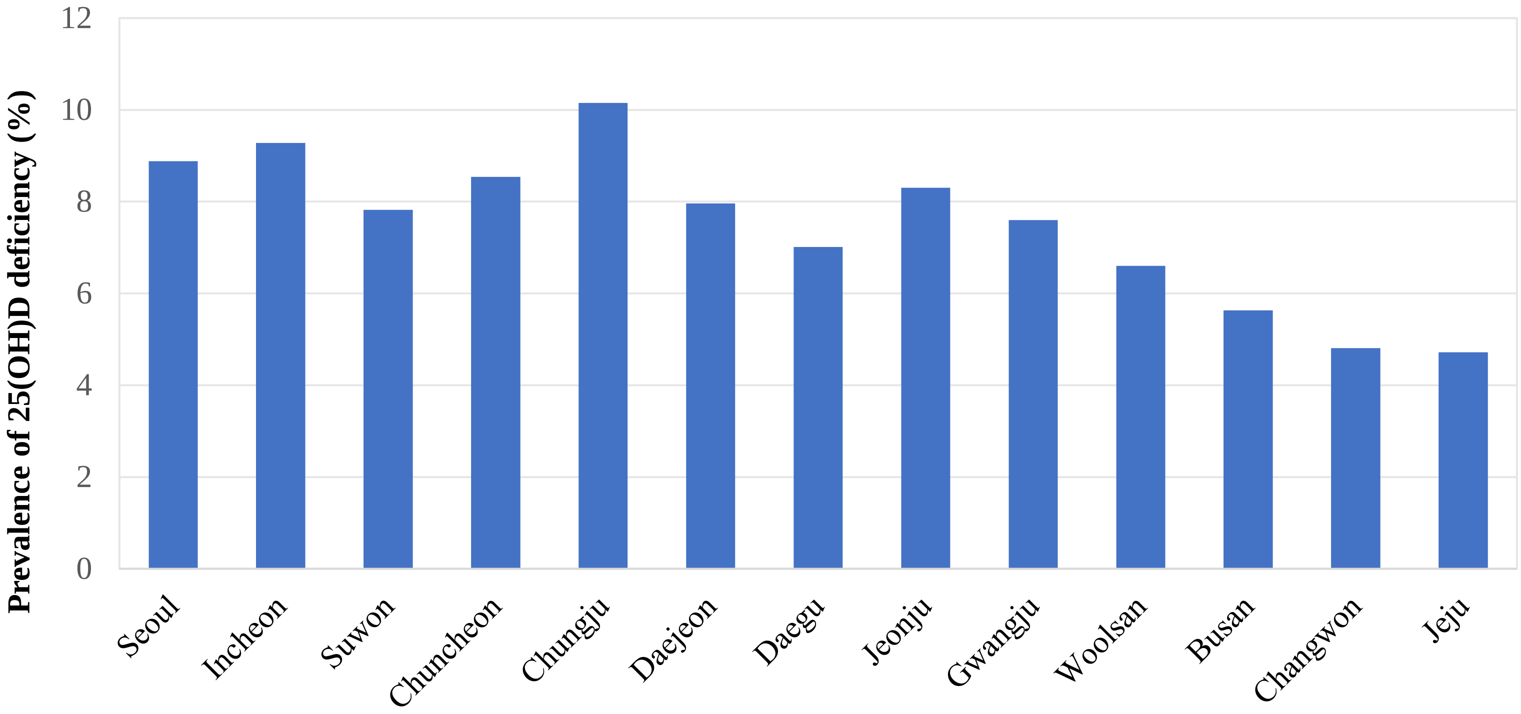

Supplementary Figure S3. Prevalence of serum 25(OH)D deficiency (<25 nmol/L) by city of residence. 25(OH)D, 25-hydroxyvitamin D

Supplementary Table S1. Detection rate of $25(OH)D_2$ by sex and age

	$25(OH)D_2$ detected	
	N	%
Total	11,024	9.2
Sex		
Male	4,681	8.0
Female	6,343	10.3
Age		
20-29	136	6.8
30-39	704	7.6
40-49	1,674	7.5
50-59	3,375	8.9
60-69	3,990	10.6
Over 70	1,145/	11.1

25(OH)D, 25-hydroxyvitamin D
